# Evaluation of Mobile Applications for Patients with Diabetes Mellitus: A Scoping Review

**DOI:** 10.3390/healthcare12030368

**Published:** 2024-01-31

**Authors:** Jung Lim Lee, Youngji Kim

**Affiliations:** 1 Department of Nursing, Daejeon University, Daejeon 34520, Republic of Korea; lleejl@dju.kr; 2 Department of Nursing, College of Nursing and Health, Kongju National University, Gongju 32588, Republic of Korea

**Keywords:** diabetes, mobile applications, review, telemedicine

## Abstract

There has been increasing interest in mobile healthcare for diabetes management. However, there remains limited evidence regarding the effectiveness of these mobile applications (apps). This scoping review aimed to evaluate the clinical effectiveness of mobile diabetes management apps. We used the following search terms: “mobile app”, “mobile application”, and “diabetes”. We included only articles written in English and published between January 2016 and August 2021. We identified two, six, and four articles focused on type 1 diabetes, type 2 diabetes, and both diabetes types, respectively. Five, four, and three of these studies reported on the apps’ functionality, usability, and both, respectively. Our findings indicated that diabetes mobile apps allowed for convenient user experience and improved blood sugar levels in patients with diabetes. Considering these findings, usability must be comprehensively evaluated by using definitions such as the ISO9241-11 usability definition or the mobile application rating scale (MARS) when developing diabetes-related apps. For the feasibility of diabetes mobile apps, we recommend that HbA1C and self-management be included as evaluation variables. Given the increasing importance of continuous management for patients with diabetes, interventions using mobile apps are bound to become effective tools for patient-led self-management.

## 1. Introduction

Diabetes mellitus is among the fastest growing global health issues. According to the International Diabetes Federation Diabetes Atlas, there were 537 million adults aged 20–79 years with diabetes worldwide in 2021, with this number being projected to increase by 46% by 2045. Additionally, there were >1.2 million children and adolescents with type 1 diabetes in 2021, with this number showing an annual increase [1]. Diabetes leads to serious complications such as blindness, kidney failure, heart attacks, stroke, and lower limb amputation [2]. In 2021, diabetes or its complications accounted for 12% of all-cause deaths worldwide among adults aged 20–79 years; moreover, 32.6% of diabetes-related deaths occurred in people aged <60 years [1]. Additionally, 24.5% of adults with diabetes achieved glycosylated hemoglobin (HbA1C) <6.5%, with only 9.7% of them achieving glycemic, blood pressure, and lipid control [3]. Accordingly, there is an urgent need for effective diabetes management strategies.

The long-term health outcomes of patients with diabetes can be improved through appropriate self-management activities, including walking exercise, diet, smoking cessation, and glucose monitoring [2,4]. Mobile healthcare has spread with advances in information and communications technology and the increased supply of smartphones [5], especially with the coronavirus disease 2019 (COVID-19) pandemic [6]. According to the IQVIA Digital Health Trends 2021, >350,000 digital health applications (apps) and >90,000 new apps were released in 2020 [6]. Unlike clinic- or hospital-provided diabetes education, mobile apps are accessible without time and space restrictions [7]. Moreover, mobile apps meet various user needs, elicit user interest through various methods such as games, and provide timely feedback [8,9,10]. With the introduction of an integrated input method, there is increasing use of mobile apps for diabetes management [5].

Diabetes management apps with various functions have been developed. Some diabetes management apps allow automatic and wireless data transfer from the measurement device via Bluetooth to a mobile device [11]. Most diabetes management apps have documentation and analysis functionality, which allows for the recording and analysis of various parameters, including blood glucose, eating habits, physical activity, or medical therapy, allowing users to track the disease course [10,11,12]. Moreover, the apps provide an advisory function or therapeutic support based on recorded data. They also have data forwarding and communication functions [11,12]. Mobile diabetes management apps have significantly facilitated HbA1C reduction [8,13] and improved medication adherence [14]. Additionally, they have significantly improved self-management activities, skills, and self-efficacy [8,15].

Given the increasing research on mobile apps for diabetes management, there have been several systematic literature reviews on the functions, effectiveness, and usability of these mobile apps [8,11,16]. However, they mainly focus on specific app-related content. To inform the development of mobile apps that sufficiently meet user needs, we aimed to perform a scoping review of studies on diabetes management apps published in the past five years. Specifically, this review focused on the functions and effectiveness of diabetes management apps, which could inform their future development and application.

## 2. Materials and Methods

This scoping review evaluated mobile apps for diabetes management. It comprised five steps: (1) identifying the research question; (2) identifying relevant studies; (3) study selection; (4) charting the data; and (5) collating, summarizing, and reporting the results.

### 2.1. Identifying the Research Question

The research question was formulated according to the components of the core question using the Population-Concept-Context framework: the population was patients with diabetes, the concept was the evaluation of diabetes management apps, and no restrictions were imposed regarding context [17]. Specifically, the research question was formulated as follows: “What does the current mHealth research on diabetes management apps reveal?”.

### 2.2. Identifying Relevant Studies

Given the increased interest in mobile apps since the “Fourth Industrial Revolution” was first discussed at the Davos Forum in 2016, we targeted academic journal articles on diabetes management apps published from 2016 to August 2021. A literature search was conducted from 26 August 2021 to 25 September 2021 in the following databases: PubMed, Cumulative Index of Nursing and Allied Health Literature (CINAHL), Database Periodical Information Academic (DBpia), and Research Information Sharing Service (RISS). The search terms included “diabetes mellitus”, “mobile application”, “mobile app”, and “evaluation”. The process of developing the query for our systematic literature review was initiated through iterative research, with a focus on two primary class topics: “diabetes” and “mobile applications”. For example, the search string used for the database query in PubMed was as follows: (((diabetes) OR (diabetes mellitus)) OR (DM)) AND (((mobile) OR (app*)) OR (mobile app*)). The languages were limited to Korean and English. We excluded conference posters, abstracts, and books, as well as studies that did not include patients with diabetes.

### 2.3. Study Selection

The identified articles were independently reviewed by two researchers. In cases of disagreement, a consensus was reached through mediation by the principal investigator. A total of 720 articles were identified, including 495, 219, 3, and 3 articles in PubMed, CINAHL, DBpia, and RISS, respectively. After excluding duplicates, the titles and abstracts of 662 articles were reviewed. After excluding 616 papers that did not meet the selection criteria in terms of research purpose, participants, and content, 46 papers were initially selected. The inclusion criteria were as follows: the articles must be written in English, and they must be human studies that objectively evaluate diabetes mobile apps. There were no restrictions on the research design. Participants were patients with diabetes—either type 1, type 2, or both. The excluded studies mainly involved animal, genetic, biochemical, and molecular biology research; furthermore, they did not use a mobile app as an intervention tool. Articles that did not specifically reveal the effect of the diabetes mobile apps, or did not deal with diabetes or mobile apps, were excluded.

**Figure 1:**
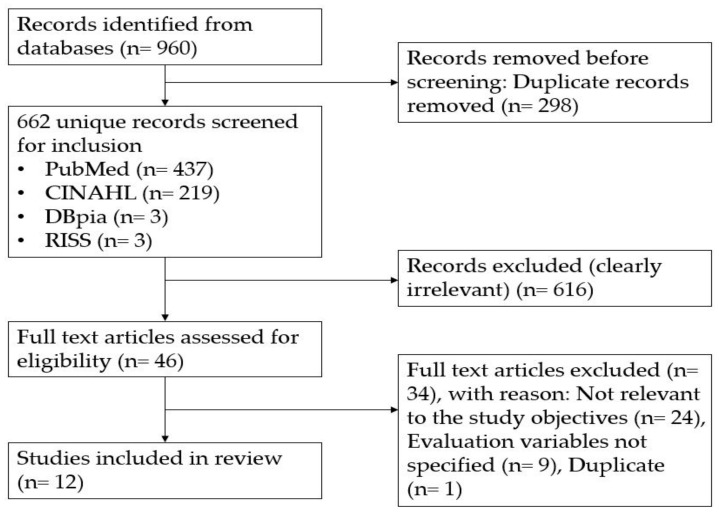
Flow diagram.

Subsequently, a full-text review of the remaining articles was independently performed by the researchers to select eligible articles, with disagreements being resolved through a consensus meeting with the principal investigator (Cohen’s kappa = 0.96). Accordingly, 34 papers were excluded, with 12 papers finally being included. The excluded studies did not evaluate mobile apps, specify evaluation variables, or mainly focus on user experiences. Figure 1 shows the data collection, selection, and extraction processes.

### 2.4. Charting the Data

Data charting refers to data extraction in a scoping review. The two researchers used a data entry form to extract relevant data from the selected literature. The extracted data comprised general information about the study (country, target group, sample size, and duration of intervention), and specific information related to the research question (app-related information, app description, evaluation type, measured outcomes, and results).

### 2.5. Collating, Summarizing, and Reporting the Results

The analysis and summary of the results are provided in the following tables according to the key themes. We performed a descriptive and numerical summary of the collected general information as well as a thematic construction process for the collected specific information (Table 1). Table 2 and Table S1 present information regarding the diabetes management apps, including the app name, platform, app development status and guidelines, and app-specific functions. Studies that included users, usability, and satisfaction as measurement variables were classified as “usability”; studies that included “hypoglycemia”, “HbA1C”, and “serum blood glucose” as measurement variables were classified as “functionality”; and studies that covered both areas were classified as “all applicable” (Table 2 and Table S1).

## 3. Results

### 3.1. General Information Regarding the Studies

#### 3.1.1. Year of Publication and Country of Study Conduct

Among the twelve included papers, two (16.7%), one (8.3%), seven (58.3%), and two (16.7%) articles were published in 2017, 2018, 2019, and 2020, respectively. The studies were conducted in ten countries; specifically, one study each (8.3%) was conducted in Iran, the United States, Denmark, New Zealand, Korea, Sri Lanka, Spain, and Canada, while two studies each (16.7%) were conducted in Australia and Singapore.

#### 3.1.2. Participants and Sample Size

There were eleven papers (91.7%) on adults and one paper (8.3%) on adolescents. The diabetes type was type 1, type 2, and both in two (16.7%), six (50.0%), and four (33.3%) articles, respectively. The number of participants ranged from 8 to 215.

#### 3.1.3. Intervention Duration

The intervention periods were ≤4 weeks, 12 weeks, and 4–12 months in five (41.2%), one (16.7%), and six (50.0%) articles, respectively.

### 3.2. Diabetes Mobile Apps

#### 3.2.1. App Name and Development Platform

The included studies examined the following apps: Gamelet, DIABETEYAR, MyT1Dhero, Medisafe, Switch, Intelligent Diabetes Management, BetaMe/Melon, Smart Glucose Manager, BlueStar mobile, SocialDiabetes app, and My Care Hub. The app name was not determined in one study (S4) since it was a prototype app. The app platforms were Android only, iOS only, both Android and iOS, and unspecified in five (S2, S3, S6, S11, S12), two (S4, S5), four (S1, S7, S8, S9), and one (S10) articles, respectively.

#### 3.2.2. Development Status and Development-Related Guidelines

Three studies (S1, S3, S4) directly developed the app and described the basis for app development. S1 developed the app contents using the recommendations of the American Diabetes Association (ADA), the organization’s guidelines, and experts’ experience. S3 developed the app based on information collected through focus groups and interviews, as well as from the latest literature. S4 developed the app based on evidence-based clinical guidelines. The remaining ten apps had already been developed (Table 2).

#### 3.2.3. App Functions

Among the twelve apps, nine provided a function for inputting health parameters, including blood sugar and psychological data, as well as a self-management function for inputting and monitoring data, including diet, medication, and exercise. Five apps provided a recommendation function, while three apps provided education. Two apps provided a reminder function and one app provided goal-tracking and communication platform functions (Table 2). Gamelet and the apps used in S4 were operated as games.

#### 3.2.4. Evaluation of Diabetes Management Apps

The apps were evaluated based on usability and/or functionality. Usability mainly referred to app use and convenience, with the measurement variables being the usability index, convenience, acceptability, and satisfaction (S1, S2, S4–S6). Functionality included physiological variables such as HbA1C, blood sugar level, body mass index, and lipid profile (S7–S10). Three studies (S3, S11, S12) evaluated both the usefulness and functionality (Table S1).

The five studies (S1, S2, S4–S6) that evaluated usability reported significant system usability, ease of use, positive attitude toward use, continued intention to use, and satisfaction. Three (S8–S10) of the four studies (S7–S10) that only evaluated functionality reported a significant decrease in the A1C level after using the mobile app. Among the three studies (S3, S11, S12) that evaluated both functionality and usability, one (S11) study observed a significant decrease in A1C levels and improvement of self-management. Contrastingly, the S12 study found that the blood sugar reduction effect was not significant; moreover, mobile app use did not significantly influence self-efficacy, quality of life, or medical service use behaviors. The S3 study investigated conflicts with family members, with the main outcome variables being family dynamics and support. Three studies (S3, S11, S12) evaluated usability in terms of satisfaction, ease of use, and app usage. Six (S7–S12) and four (S4, S6, S8, S12) studies included biomarkers (HbA1C, serum blood glucose, and fasting plasma glucose) and self-management, respectively (Table S1).

## 4. Discussion

This scoping review evaluated the basis for app development, functions, operation methods, and effectiveness of diabetes management apps by analyzing twelve related studies published over the past five years. Our findings could inform future directions for the development and use of mobile applications.

Among the twelve included studies, six and two studies included patients with type 2 and type 1 diabetes, respectively. This could be attributed to the fact that 98% of adult patients with diabetes have type 2 diabetes, with eleven of the included studies having been conducted on adults [25]. However, type 1 diabetes places an enormous burden on society and is expected to rapidly increase since its onset occurs at a young age, leading to long-term complications, shortened life expectancy, decreased quality of life, and increased individual and national medical costs [26]. Wang et al. [8] conducted a systematic review and meta-analysis of randomized control trials on the use of mobile health for type 1 diabetes management. Among the eight included studies, three studies included participants aged <20 years. Children with type 1 diabetes require lifelong insulin treatment and self-management; therefore, there is an urgent need for the active development and use of mobile apps for young patients with type 1 diabetes.

In the present review, three studies that reported the development of an app described the basis for app development. However, only one study (S1) specifically described the evidence source as being based on the ADA guidelines, while the other two studies (S3, S4) collected information from evidence-based clinical guidelines, current literature, focus groups, and interviews. Accordingly, it was difficult to ascertain the evidence sources accurately. Therefore, when developing an app, it is necessary to specify that the content is based on the latest evidence. Additionally, although there remains no standard method for evaluating app development, the involvement of users, developers, and clinical experts is considered essential during the app development stage [27]. It was difficult to confirm whether the app development in the included studies involved users, developers, and clinical experts. Moreover, only one study (S3) considered feedback from key stakeholders through focus groups and interviews in the app development process. Additionally, none of the included studies considered information security and privacy protection at the app development stage, which is a major element of digital health evaluation [28]. Taken together, there is a need to establish guidelines or standard instructions for app development.

The most included functions in the diabetes management apps were inputting health indicators such as blood sugar and self-management (diet, exercise, and medication), while the least included functions were tracking, reminders, and communication platforms. The main functions were commonly incorporated in mobile apps for adults with type 1 and type 2 diabetes. Similarly to the diabetes management apps, a mobile app for hemodialysis patients included functions for diet and weight management as well as numerous one-way inputs [20,29]. Furthermore, a previous study reported that having a complication prevention module in app-based interventions was associated with a greater HbA1C reduction [30]. Complication prevention was not included in the app functions included in our twelve studies, and some complications were included in the education module. It is necessary to consider adding complication prevention items to mobile app design for type 1 and 2 diabetes management in the future.

In this study, there was only one study (S3) targeting type 1 adolescents and their parents. Apps for adolescents and parents were developed separately and utilized as a communication platform. According to a systematic review of mobile apps for children and adolescents with cancer and their families, the main app modules were symptom assessment (90%), provision of information and education (74%), communication with caregiver (57%), social support including peer support (30%), and calendar and reminder (21%) [31]. Moreover, in a study on online interventions for young drug users, the key recommendations presented by 90 international experts for the development and implementation of online intervention were technical aspects, interactive elements, reaching young drug users, motivation to use the intervention, and evaluation [32]. The most critical issues for young drug users were design and functionality, presence of a clear structure, comprehensive and quick professional feedback, data security, playful elements, and the ability to share [32]. Based on these findings, it is necessary to add communication, social support (e.g., peer support), and entertainment elements to the disease management in online interventions for children and adolescents.

The communication function shows that mobile apps can evolve from a one-way method to an interactive method; accordingly, it is considered as an important feature to pursue in future app development. To increase the usability and utility of apps, the frequency of app use must be increased; however, a recent study reported a decreasing trend in the frequency of app use over time [20]. Accordingly, to increase the frequency of app use, clinical experts should actively recommend app use or advocate for it as a medical aid tool. Specifically, to increase motivation for use, it may be feasible to include a community function between users or between the user and an attending physician or outpatient nurse. Most of the included functions involve information input and health advice; accordingly, real-time or two-way communication functions should be considered in the future. Therefore, it is important to incorporate interactive live communication that goes beyond one-way information input and preprogrammed message reception.

In our review, usability was evaluated in eight studies, and the main measures were ease of use (23.5%), app usage (23.5%), system usefulness (17.6%), and satisfaction (11.8%). The least included measures were learnability, intelligibility, perceived value, and intention and behavior towards recommendation. In a study that classified the attributes of 790 documents for mobile apps from 2001 to 2018 according to the ISO9241-11 usability definition, the most commonly used attributes were efficiency (70%), satisfaction (66%), and effectiveness (58%) [33]. Other attributes were learnability (45%), memorability (23%), cognitive load (19%), errors (17%), simplicity (13%), and ease of use (9%) [33]. Among the attributes used in this study, only satisfaction was evaluated among the ISO9241-11 usability definitions, and efficiency (duration spent on each screen, duration to complete task, user’s error rate) and effectiveness (number of successfully completed tasks, number of steps required to complete tasks, etc.) were not evaluated. In addition, in a study that evaluated 63 COVID-19 apps using the mobile application rating scale (MARS), the overall app quality (engagement, functionality, aesthetics, information) showed high functionality and low engagement [22]. In the results of this study, only functionality was measured, and other factors were not measured. Considering these findings, it is proposed to comprehensively evaluate usability by using definitions such as the ISO9241-11 usability definition or MARS when developing diabetes-related apps.

Moreover, seven studies that evaluated functionality showed that the mobile apps were most effective in terms of blood sugar reduction. In a meta-analysis of twenty-two studies on mobile app-based interventions for chronic pain, the primary outcome was pain [34], and when applying a mobile app for child and adolescent mental health, the main outcomes were mood change, emotional response, and self-awareness [35]. Therefore, measuring HbA1C as the primary outcome in the diabetes app was validated. Specifically, app use led to a significant decrease in the HbA1C level by 0.6%, 0.3%, 1.3%, and 0.9% in S8, S9, S10, and S11, respectively. The S7 study did not report HbA1C levels; moreover, in the S12 study, the mean difference was −0.42, which was not statistically significant. Some studies have shown that smartphone apps can help people with type 1 and 2 diabetes improve and reduce their levels of HbA1C [36,37,38] while other studies for type 1 diabetes have reported a low clinical impact of app use on HbA1C levels [39,40]. These inconsistent findings can be attributed to differences in study participants, application times, and app usage frequencies. In this study, three of the twenty-five studies that measured HbA1C were randomized controlled trials (RCTs). For the validity of the results, more RCT studies on the effectiveness of apps should be conducted, and the results of the conducted studies should be integrated.

The secondary outcomes in this study were the self-appraisal of diabetes, self-management level, barriers to medication adherence, experience of care, diabetes family conflict and general tone of family communication, and self-reported health utilization. To effectively manage blood sugar levels, it is necessary to also emphasize self-management functions. The use of apps that comprise self-management functions (healthy eating, activity, monitoring, medication, risk reduction, problem solving, and healthy coping) has been shown to improve the daily lives of patients with diabetes [41]. Additionally, the use of a diabetes management app developed with the goal of improving self-management was shown to improve HbA1C levels and self-efficacy in patients with diabetes [42]. Including the self-care function in a diabetes management app is considered to improve its functionality [43,44]. In a study using a mobile app for type 1 children and adolescents, the prevalence of hypoglycemia and quality of life were measured as secondary outcomes, and although there was a significant difference in the frequency of hypoglycemia, there was no significant difference in quality of life [36]. In addition, from a long-term perspective, indicators such as healthcare service utilization and the prevalence of complications should be measured for the effectiveness of apps [45].

This study contributes to the future development of mobile apps by conducting an in-depth analysis of mobile apps, which are powerful tools that can assist patients with diabetes, a representative chronic disease, in self-management. Our findings describe the research trends related to diabetes management apps and may inform strategies for developing mobile apps that improve self-management among patients with diabetes as well as future studies.

Only one of the twelve studies was conducted on adolescents, and it focused on communication between parents and children rather than on overall health management, making it impossible to state that it reflects the overall evaluation of mobile apps. In the future, a thorough examination of studies aimed at adolescents is required to investigate the usefulness of mobile apps for this age group. We believe that there can be a real risk of losing relevant information when focusing on keywords and choosing a narrow search for a PubMed search. Therefore, we suggest introducing several simulations before deciding on the search formula [46].

None of the studies we reviewed aimed to address the integration of diabetes mellitus mobile apps in routine clinical practice as part of their treatment regimen. In the future, researchers should present a framework for prescription apps to address the problem of the lack of embedding of mobile apps in routine care.

## 5. Conclusions

This study sought to compile information from previously published articles by succinctly summarizing the beneficial effects of mobile apps for managing diabetes. Our findings indicated that diabetes mobile apps allowed convenient user experience and improved blood sugar levels in patients with diabetes. Considering these findings, we propose to comprehensively evaluate usability by using definitions such as the ISO9241-11 usability definition or MARS when developing diabetes-related apps. For the feasibility of diabetes mobile apps, HbA1C and self-management should be included as evaluation variables, and more RCT studies on the effectiveness of apps should be conducted. Given the increasing importance of continuous management for patients with chronic diseases, nursing interventions using mobile apps are bound to become effective tools for patient-led self-management, such as blood sugar control in patients with diabetes.

## Figures and Tables

**Table 1:** Summary of evaluation studies on diabetes mobile applications.

Study Code	Author	Country	Target Group	Sample Size	Duration of Intervention
S1	Kho et al., 2019 [9]	Singapore	Type 2 DM with HbA1C >7%	8	2 weeks
S2	Janatkhah et al., 2019 [12]	Iran	Type 1 and 2 DM adults	136	2 weeks
S3	Holtz et al., 2019 [18]	USA	Type 1 DM adolescents with their parents	10	4 weeks
S4	Peterson and Hempler, 2017 [19]	Denmark	Newly diagnosed type 2 DM	14	4 weeks
S5	Signal et al., 2020 [20]	New Zealand	Pre-diabetes or type 2 DM adults with HbA1C 41–70 mmol/mol	215	12 months
S6	Adu et al., 2020 [15]	Australia	Type 1 and 2 DM adults having a current recommended blood glucose level (BGL) target of 4–10 mmol/L	41	3 weeks
S7	Huang et al., 2019 [14]	Singapore	Type 2 DM, at or above the age of 21 years	41	12 weeks
S8	Lee et al., 2018 [21]	South Korea	Type 2 DM with HbA1C >6.5%, ≥19 years	158	phase I: 6 months, phase II: 6 months
S9	Ryan et al., 2017 [22]	Canada	Type 1 DM adults (onset of DM 11.8 ± 6.9 years)	18	observation phase of 4 weeks and a subsequent active phase of 4 months
S10	Vehi et al., 2019 [23]	Spain	Type 1 and 2 DM adults having diabetes for more than 1 year and no complication and HbA1C ≥8%	211	6 months
S11	Gunawardena et al., 2019 [13]	Sri Lanka	Type 1 and 2 DM adults with HbA1C >8%	54	6 months
S12	Agarwal et al., 2019 [24]	Canada	Type 2 DM adults with HbA1C >8%	139	6 months

**Table 2:** Functions of mobile apps for patients with diabetes.

App Name/Function	Goal Tracking	Monitoring or Recording			Health Parameter	Education	Communication Platform	Recommendation	Reminder
		Diet	Medication	Exercise					
Gamelet	□	√	√	√	□	√	□	□	□
DIABETEYAR	□	□	□	□	□	√	□	√	□
MyT1DHero	□	□	□	□	√	□	√	□	√
Medisafe	□	□	√	□	√	□	□	□	□
Switch	□	□	□	□	√	□	□	□	□
Intelligent Diabetes Management (IDM)	□	□	□	□	√	□	□	√	□
BetaMe/Melon	√	□	□	□	□	□	□	□	□
SocialDiabetes app (SDA)	□	□	□	□	√	□	□	√	□
the Smart Glucose Manager (SGM)	□	√	√	□	√	□	□	□	√
BlueStar mobile	□	√	√	□	√	□	□	√	□
My Care Hub (MCH)	□	√	□	√	√	√	□	√	□
Prototype *		√		√	√				

* Study ID: S4.

## Data Availability

Data are contained within the article.

## Supplementary Materials

The following supporting information can be downloaded at: https://www.mdpi.com/article/10.3390/healthcare12030368/s1, Table S1: Features of diabetes mobile applications in evaluation studies.
