# Supplementary material for: Evaluation of Mobile Applications for Patients with Diabetes Mellitus: A Scoping Review
Source: Healthcare (Basel). 2024 Jan 31;12(3):368. doi: 10.3390/healthcare12030368 (PMC10855494; doi:10.3390/healthcare12030368)
Supplement: Supplementary file 1 [file healthcare-12-00368-s001.zip › healthcare-2794600-supplementary.pdf]

Supplementary Table S1. Features of diabetes mobile applications in evaluation studies.

No.	Name	Platform	App. development	Development guideline	Contents or app description	Evaluation type (F/U) *	Measured outcomes	Main results
S1	Gamelet	iOS, Android	Yes	Recommendations from American Diabetes Association	Gamelet 1: good diabetes care<br>Gamelet 2: hypoglycemia management	U	1) System usefulness<br>2) Perceived ease of use<br>(21 items, 5-point Likert scale)	1) System usefulness, 4.7±0.32<br>2) Perceived ease of use, 4.4±0.57
S2	DIABETEYAR	Android	No	NA	1) Physician recommendation<br>2) Guidelines for safe exercise, sports tips<br>3) Diet recommendations<br>4) Blood glucose notes<br>5) Capture photos of DM complication	U	The modified Telehealth Usability Questionnaire (TUQ)<br>1) Usefulness<br>2) Ease of use and learnability	1) Usefulness, 4.57±0.5<br>2) Ease of use and learnability, 4.78±0.4
S3	MyT1DHero	Android	Yes	1) Information from focus groups and interviews<br>2) Current literature	1) Customizable blood glucose reminders and ranges,<br>(1) parent app: view their child's blood glucose trends and communicate with their child, (2) child app: log their blood glucose and send and view their blood glucose	F/U	1) 3 items from the Diabetes Family Conflict Scale (DFCS)<br>2) 4 items, general tone of family communication<br>3) Satisfaction<br>4) Perception of ease of use<br>(5-point Likert scale)	1) Significant decrease of conflict: parents (results of blood sugar monitoring and logging blood sugar results), adolescent (logging blood sugar results)<br>2) The MyT1DHero app was understandable, 4.10±0.97<br>3) It was easy to become skilled at using the app, 4.00±1.08
S4	NR	iOS	Yes	Evidence based clinical guideline	5 functions:<br>1) Overview of diabetes activities<br>2) Recording of health data,<br>3) Reflection games and goal setting<br>4) Knowledge games<br>5) Recording of psychological data such as sleep, fatigue, and well-being	U	Themes identified in the implementation phase:<br>1) A viable tool to support diabetes self-management,<br>2) Patterns of app use	1) Users found the app to be a valuable tool for support, particularly for raising their awareness about their psychological health and for informing and guiding them through the healthcare system after diagnosis.<br>2) The two most frequently used app functions were "My health data," in which users could record health data such as blood sugar and HbA1C, and "Status," which allowed them to record data related to well-being, sleep, ad stress.

S5	BetaMe/Melon	iOS	No	NA	1) Health coaching,<br>2) Health literacy component,<br>3) Goal tracking,<br>4) Peer support	U	1) Utilization pattern from the mobile/web platform,<br>2) The usefulness of the program and recommendation to others from online survey	1) Out of the 215 participants, 198 (92%) received an initial health coaching session, and 160 (74%) were actively engaged with the program at some point during the 16-week core program. Usage steadily declined from 50% at Week 0 to 23% at Week 15.<br>2) Participants ranked component usefulness as education resources (63.7%), health coaches (59.2%), goal tracking (48.8%), and online peer support (42.1%). Although 53% agreed that the program was easy to use, 64% would recommend the program to others.
S6	My Care Hub (MCH)	Android	No	NA	1) "Documentation" and "Analytic" features: to monitor BGLs, physical activities, and food intake<br>2) Education models: (1) "Information" on portion sizes of each food, (2) "Motivation"	U	1) Preliminary efficacy: diabetes self-management activities (10 items, the Summary of Diabetes Self-Care Activity (SDSCA) questionnaire), skills, and self-efficacy (17 items, LMC Skills, Confidence and Preparedness Index (SCPI)),<br>2) the app's acceptability: 5 Likert scale, ease of use, intelligibility, satisfaction, perceived value, intention, and behavior towards recommendation	1) Statistically significant improvements were observed between pre- and post-intervention measures: DSM activities (4.55 ± 1.14 vs. 5.35 ± 0.84; $p = 0.001$); skills (7.10 ± 1.99 vs. 7.90 ± 1.67; $p = 0.04$); and self-efficacy (7.33 ±1.83 vs. 8.07 ± 1.54; $p = 0.03$).<br>2) Overall mean ratings for all of the items were above 3 on the 5-point scale; suggesting that participants were satisfied with the app's ease of use and educational content.
S7	Medisafe	iOS,<br>Android	No	NA, commercial, free medication app	1) Medication scheduling, reminder, tracking, data sharing<br>2) Medication adherence assessments	F	1) Feasibility: (1) recruitment/enrolment rate, (2) adherence to trial participation,	1) A total of 176 patients were recruited. 51 (31.7%) participants met the inclusion criteria and were enrolled in this study. 41 participants completed the postintervention survey.

							2) Effectiveness: (1) barriers to medication adherence (Adherence Starts with Knowledge-12, ASK-12), (2) self-appraisal of diabetes (Appraisal of Diabetes Scale, ADS)	2) The mean ASK-12(adherence barrier) score was significantly lower in the intervention group than in the control group (mean difference: 4.7, p=.01). There were no statistically significant mean differences between groups in ADS score.
S8	Switch	iOS, Android	No	NA	1) Upload their lifestyle and medical information<br>2) Information on diabetes and other metabolic diseases	F	1) Effectiveness: (1) clinical data: HbA1C, fasting plasma glucose (FPG), total cholesterol (TC), triglycerides (TG), high-density and low-density lipoprotein cholesterol (HDL-C and LDL-C)<br>2) Self-management level and diabetes awareness: the Korean version of the Summary of Diabetes Self-Care Activities Questionnaire (SDSCA) and the Korean version of the Appraisal of Diabetes Scale (ADS)	1) Phase I result: (1) HbA1C levels significantly decreased in only I-M group (0.6%) (2) SDSCA: significant improvement, especially in exercise frequency, SMBG, the number of cigarettes in the I-M group only and foot care in both groups (3) ADS: significant improvement in the sense of self-control in the I-M group only<br>2) Phase II: (1) HbA1C levels of the C-I group who received TMC during phase 2 of the study decreased by 0.6% compared to phase 1 levels. In the I-M group, initial improvement in HbA1C levels at three months continued until 12 months. (2) SDSCA: frequency of exercise and SMBG in the C-I group significantly increased during the intervention period. The frequency of utilizing a general diet in the C-I group and a special diet in the I-M group increased.
S9	Intelligent Diabetes Management (IDM)	iOS, Android	No	NA	1) To record individual glucose levels<br>2) To propose carbohydrate intakes and planned activities<br>3) To suggest the appropriate insulin doses<br>4) To link to a website, Intelligent Diabetes Management (IDM)	F	1) Primary outcome: changes in HbA1C levels.<br>2) Secondary outcomes: (1) investigators' time spent reviewing records, (2) mean glucose levels at pre-meal times	1) Primary outcome: The median A1C level fell from 8.1% (7.5 to 9.0, IQ range) to 7.8% (6.9 to 8.3; p<0.001)<br>2) Secondary outcomes: (1) investigators' time spent reviewing records in the active phase, review of the glucose diaries took less time on the IDM website than using personal glucose records in the observation period, median 6 minutes (5 to 7.5 IQ range) vs. 10 minutes (7.5 to 10.5 IQ range; p<0.05).

S10	SocialDiabetes app (SDA)	NR	No	NA	1) Remote monitoring of patients<br>2) Personalized insulin dose recommendations<br>3) HbA1c estimation<br>4) Charts & insights<br>5) Carbohydrate calculator & meal planning<br>6) Connection with healthcare professionals,<br>7) Alerts & reminders	F	1) Estimated HbA1c,<br>2) Changes in HBGI and LBGI	1) A reduction of estimated A1c (eA1c) of approximately 1.3% (p<.001) and 0.9% (p=.001) for Type 1 DM groups A and B, respectively, and 2% (p<.001) for both A and B Type 2 DM groups, respectively<br>2) Type 1 DM baseline LBGI values for groups A and B were 5.2 (SD 3.9) and 4.4 (SD 2.3), respectively, which decreased at t2 to 3.4 (SD 3.3) and 3.4 (SD 1.9), respectively; this was a reduction of 34.6% (p=.005) and 22.7% (p=.02), respectively. Baseline HBGI values for groups A and B were 12.6 (SD 4.3) and 10.6 (SD 4.03), respectively, which decreased at t2 to 9.0 (SD 6.5) and 8.6 (SD 4.7), respectively; this was a reduction of 30% (p=.001) and 22% (p=.003), respectively.
S11	The Smart Glucose Manager (SGM)	Android	No	NA	1) Reminders (check blood glucose, take medication on time, eat on time, exercise at use-defined times)<br>2) Glucose entry<br>3) Profile<br>4) Meds and insulin<br>5) Calculator<br>6) Logbook and graph	F/U	1) Primary outcome: changes in A1C levels<br>2) Secondary outcomes: SGM usage	1) At the six-month follow up, the SGM group had significant lower A1c levels than the control group (7.2% vs 8.17%, p<.0001).<br>2) For both groups, A1c values decreased from baseline to three months (SGM: 9.52% to 8.16%, p<.0001; control: 9.44% to 8.31%, p<.0001). From the third to sixth month, the SGM group showed further improvement of A1c (−0.96%, p<.0001), whereas the control group did not (p=0.19). A1c improvement was positively correlated with SGM usage (R=.81, p<.001).
S12	BlueStar mobile	Android	No	NA	A virtual coach for patients:<br>1) diabetes management,<br>2) to provide tailored messaging to "coach" participants	F/U	1) Primary outcome: HbA1C levels at three months<br>2) Secondary outcomes: (1) patient self-management (Problem Areas in Diabetes & Summary of Diabetes Self-Care Activities), (2) experience of care (EuroQol-5D), (3) self-reported health utilization,	1) The results of an analysis of covariance controlling for baseline HbA1C levels did not show evidence of intervention impact on HbA1C levels at 3 months (mean difference [ITG−WLC] −0.42, 95% CI −1.05 to 0.21; P=.19).<br>2) Similarly, there was no intervention effect on secondary outcomes measuring diabetes self-efficacy, quality of life, and health care utilization behaviors.

	3) Intervention usage	

F/U, functionality/usability; TMC, tailored mobile coaching; I-M, Intervention-Maintenance; C-I, Control-Intervention; ITG-WLC, immediate treatment group- wait-list control.
